# Protein or no protein? Using PCR for detecting allergens in foods

**DOI:** 10.1186/2045-7022-1-S1-S81

**Published:** 2011-08-12

**Authors:** Martin Röder, Stefan Vieths, Thomas Holzhauser

**Affiliations:** 1 Paul-Ehrlich-Institut, Langen, Germany

## 

The latest amendment (2007/68/EC) of annex IIIa of the European Directive 2000/13/EC currently requires 14 groups of allergenic food ingredients to be mandatory labelled. The labelling does not refer to a certain compound of an allergenic food but the allergenic food itself. Thus, every method that has been demonstrated to reliably detect the target food is applicable. State of the art in the detection of allergenic foods has been the protein based Enzyme-Linked Immuno Sorbent Assay (ELISA) and meanwhile, numerous studies have demonstrated good correlation between protein based ELISA and DNA based Polymerase Chain Reaction (PCR) methods. Even in protein enriched isolates or concentrates DNA has been proven to be an alternative molecular marker for the presence of an allergenic food. Since 2005 the number of scientific publications on PCR for allergen detection has increased tremendously, of which real-time PCR with sequence specific fluorescent probes is considered state-of-the-art technology. Both ELISA and PCR exhibit strengths and limitations: ELISA are considered a quantitative methodology with high sensitivity at a level of 1-10 mg/kg. However, known cross-reactivity to phylogenetically closely related foods or ingredients thereof may lead to false-positive results. By contrast PCR offers unparalleled specificity to avoid complaints or potentially expensive food recalls due to false-positives. For PCR, a sensitivitiy below 10 mg/kg, which is considered sufficient in comparison to known clinical threshold data, is feasible. Moreover, real-time PCR allows multi-allergen screening in one run. In addition, first PCR methods with quantitative features have been published and more are expected in the near future. Thus, PCR may complement or even substitute ELISA depending on the allergenic food to detect.

